# Friction behavior of the wire material Gummetal®

**DOI:** 10.1007/s00056-021-00317-y

**Published:** 2021-07-06

**Authors:** Isabel Eri Kopsahilis, Dieter Drescher

**Affiliations:** Department of Orthodontics, University of Düsseldorf, Düsseldorf, Germany

**Keywords:** Orthodontic brackets, Orthodontic wires, Orthodontic appliances, Tooth movement techniques, In vitro tests, Kieferorthopädische Brackets, Kieferorthopädische Drähte, Kieferorthopädische Apparaturen, Techniken der Zahnbewegung, In-vitro-Tests

## Abstract

**Objectives:**

Gummetal® (Maruemu Works, Osaka, Japan), a new orthodontic wire material successfully used in clinical applications since 2006, is biocompatible and exhibits exceptionally high elasticity, nonlinear elastic behavior, plasticity and strength. Systematic comparisons of friction behavior are lacking; thus, the friction of Gummetal® in the binding modus was compared to commonly used low friction wires.

**Materials and methods:**

In vivo tests were run with Gummetal®, CoCr (cobalt-chromium Elgiloy®, Rocky Mountain Orthodontics, Denver, CO, USA), β‑Ti (β-Titanium TMA®, Ormco, Orange, CA, USA), NiTi (nickel–titanium, NiTi-SE, Dentalline, Birkenfeld, Germany), and stainless steel (SS; Ref. 251-925, 3M Unitek, Monrovia, CA, USA) [dimensions: 0.014 inch (0.35 mm), 0.016 inch (0.40 mm), 0.016 × 0.022 inch (0.40 × 0.56 mm), and 0.019 × 0.025 inch (0.48 × 0.64 mm)—β-Ti not available in the dimension 0.014 inch]. These were combined with Discovery® (Dentaurum, Ispringen, Germany), Micro Sprint® (Forestadent, Pforzheim, Germany), Clarity™ (3M Unitek), and Inspire Ice™ (Ormco) and slots in the dimension 0.022 inch (0.56 mm) and, except for the 0.019 × 0.025 inch wires, in the dimension 0.018 inch (0.46 mm). They were ligated with a 0.010 inch (0.25 mm) steel ligature (Smile Dental, Ratingen, Germany). Brackets were angulated by applying a moment of force of 10 Nmm against the wire, which was pulled through the slot at 0.2 mm/s.

**Results:**

In 660 tests using 132 bracket–wire combinations, friction loss for Gummetal® was comparable to and, in a few combinations with Micro Sprint®, significantly lower (*p* < 0.05) than SS and CoCr. The friction for Gummetal® was significantly lower (*p* < 0.05) than NiTi, and β‑Ti. In some bracket–wire combinations, lower friction was found with round wires compared to rectangular wires, except for the combination with Inspire Ice™, which was higher but not significant. Slot size did not have a significant effect on friction in most combinations.

**Conclusion:**

The low friction associated with Gummetal® wires during arch-guided tooth movement will be a valuable addition to the armamentarium of orthodontists.

## Abstract (de)

**Zielsetzung:**

Gummetal® (Maruemu Works, Osaka, Japan), ein neues kieferorthopädisches Drahtmaterial, seit 2006 erfolgreich in der klinischen Anwendung, ist biokompatibel und weist eine außerordentlich hohe Elastizität, ein nichtlineares elastisches Verhalten sowie eine hohe Plastizität und Festigkeit auf. Systematische Vergleiche des Reibungsverhaltens fehlen; daher wurde die Reibung von Gummetal® im Bindungsmodus mit üblicherweise verwendeten Drähten mit geringer Reibung verglichen.

**Material und Methoden:**

Fünf unterschiedliche Drahtlegierungen, Gummetal®, CoCr (Elgiloy®, Rocky Mountain Orthodontics, Denver/CO, USA), β‑Ti (TMA®, Omco Corp., Orange/CA, USA), NiTi (NiTi-SE, Dentalline, Birkenfeld, Deutschland) und Edelstahl („stainless steel“, SS; 3M Unitek, Monrovia/CA, USA) in den Dimensionen 0,014 inch (0,35 mm), 0,016 inch (0,40 mm), 0,016 inch × 0,022 inch (0,40 × 0,56 mm) und 0,019 inch × 0,025 inch (0,48 × 0,64 mm) wurden in vivo miteinander verglichen – β‑Ti war in der Dimension 0.014 inch nicht verfügbar. Die Drähte wurden mit 4 konventionellen Brackets, Discovery® (Dentaurum, Ispringen, Deutschland), Micro Sprint® (Forestadent, Pforzheim, Deutschland), Clarity™ (3M Unitek, Monrovia/CA, USA) und Inspire Ice™ (Ormco Corp., Orange, CA, USA) der Slotgröße 0,022 inch (0,56 mm) und, mit Ausnahme der 0,019 × 0,025 inch Drähte, der Slotgröße 0,018 inch (0,46 mm) kombiniert. Als Ligatur wurde eine 0,010 inch (0,25 mm) Stahlligatur (Smile Dental, Ratingen, Deutschland) verwendet. Brackets wurden mit einem Drehmoment von 10 Nmm gegen den Draht anguliert, der mit 0,2 mm/s durch den Slot gezogen wurde.

**Ergebnisse:**

In 660 Tests mit 132 Bracket-Draht-Kombinationen war der Reibungsverlust für Gummetal® vergleichbar mit dem von SS und CoCr und in einigen Kombinationen mit Micro Sprint® signifikant geringer (*p* < 0,05). Die Reibung für Gummetal® war signifikant niedriger (*p* < 0,05) als NiTi, und β‑Ti. Bei einigen Bracket-Draht-Kombinationen wurde eine geringere Reibung bei runden Drähten im Vergleich zu rechteckigen Drähten festgestellt, außer bei der Kombination mit Inspire Ice™, die höher, aber nicht signifikant war. Die Slotgröße hatte bei den meisten Kombinationen keinen signifikanten Einfluss auf die Reibung.

**Schlussfolgerungen:**

Die geringe Friktion der Gummetal®-Drähte bei der bogengeführten Zahnbewegung wird eine wertvolle Ergänzung des Armamentariums der Kieferorthopäden sein.

## Introduction

For decades, various wires and brackets have been developed in an effort to improve orthodontic therapy. Many of these innovations were limited to modifying existing orthodontic materials. However, Gummetal® (Maruemu Works, Osaka, Japan), a new alloy for orthodontic wires, currently sold primarily in Japan, is of special interest since it may provide some advantages for orthodontic therapy [21, 22, 36]. It is a biocompatible β‑Ti composition of titanium, niobium, tantalum, and zirconium (Ti–23Nb–0.7Ta–2Zr–1.2O) with high strength, which can be plastically formed [21, 22]. Its Young’s modulus of about 46 GPa [22] lies between β‑Ti of about 72 GPa and NiTi of 33–44 GPa [29]. It demonstrates nonlinear elastic behavior with decreasing Young’s modulus. According to Hasegawa, who introduced Gummetal® into orthodontics and thoroughly documented its applications in clinical practice [22], its numerous properties along with the en bloc treatment strategies which he suggested [8] can optimize three-dimensional tooth movement in orthodontic therapy [22]. Hasegawa used a rectangular Gummetal® archwire immediately after leveling, without any further archwire changes which enabled good tooth control from the beginning of an orthodontic treatment [22].

Since there had been no systematic comparison of Gummetal®’s friction behavior with that of other wires, how can Gummetal®’s friction behavior be judged? Friction, defined as the force that retards or resists the relative motion of two objects in contact, has become one of the most important criteria when choosing orthodontic materials since the studies published by Andreasen and Quevedo in 1970 [3]. According to Drescher, up to 50% of orthodontic force can be lost by friction [16]. To measure the quantity of friction, numerous studies have been conducted using various combinations of orthodontic wire materials and brackets. As a result of these studies, researchers concluded that steel wires were found to have the lowest friction, particularly in connection with steel brackets [16, 46, 47]. Steel is followed by CoCr and then by NiTi [46]. β‑Ti, better known as TMA® (titanium–molybdenum alloy; Ormco Corp., Orange, CA, USA), is generally recognized as having the highest friction values [24, 30]. However, rankings may differ if, for example, combinations of specific materials are tested or the material surfaces underwent special treatment [6] as the newly introduced low-friction TMA® [2].

**Fig. 1:**
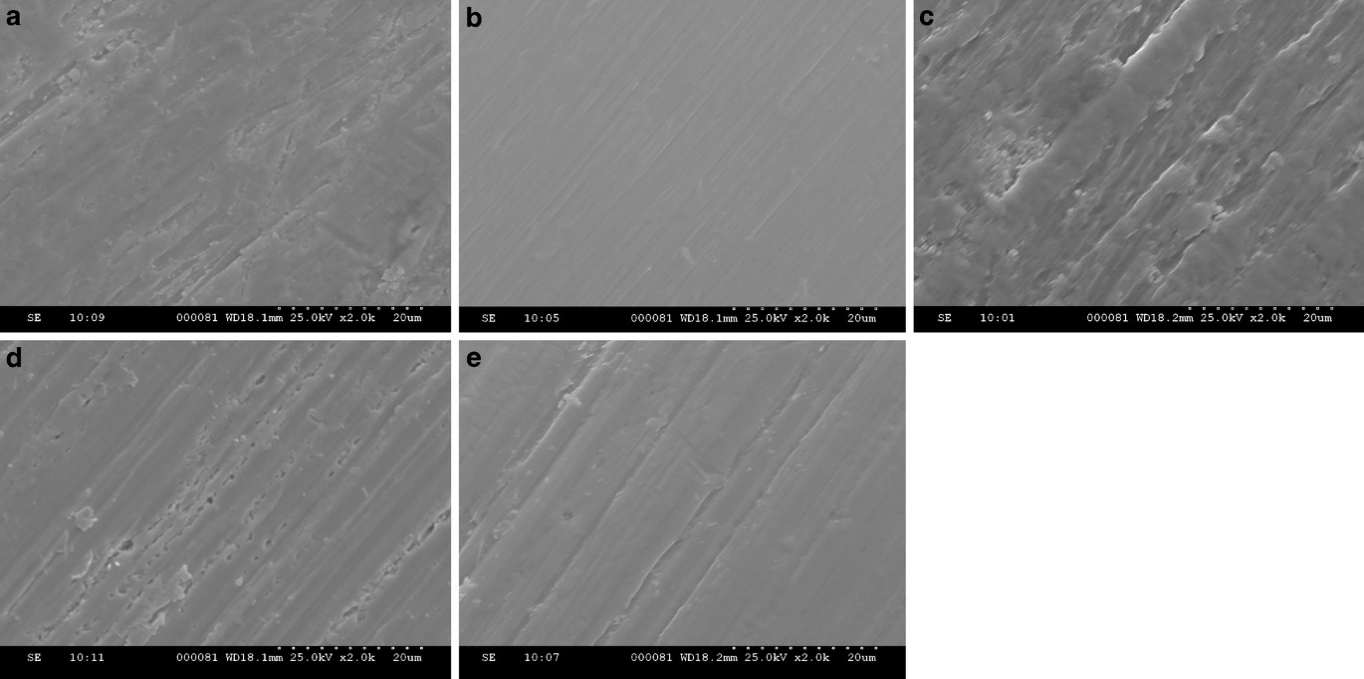
Surface structure (approx. 2000× magnification scanning electron microscope [SEM] image) of **a** Gummetal®, **b** steel, **c** β-Ti, **d** CoCr, and **e** NiTi Oberflächenstruktur (Vergr. ca. 2000:1, REM[Rasterelektronenmikroskop]-Darstellung) von **a** Gummetal®, **b** Stahl, **c** β-Ti, **d** CoCr und **e** NiTi

Friction occurs when the wire comes in forced contact with a bracket or ligature. It depends largely on the surface roughness of materials [9, 10, 15, 32, 40], as the above mentioned rankings usually coincide with the surface roughness of the wires used [10]. But, also the oral environment as well as the “frictional mode” (binding, notching, etc.) have to be considered. CoCr alloys, for instance, have smooth surfaces resulting in relatively low friction when used with metal brackets. The friction is less than that of NiTi and that of β‑Ti [16]. β‑Ti displays a distinct surface roughness [20], and frictional values are correspondingly high [16]—up to six times higher than that of stainless steel wires [15]. Scanning electron microscope (SEM) images of the surface structures of Gummetal®, stainless steel, β‑Ti, CoCr, and NiTi are shown in Fig. 1. It can be noticed that the surface of stainless steel is smooth, whereas the surface of Gummetal® appears rough.

Other factors which influence friction are the shape of the wires and their dimension. Rectangular wires typically produce higher friction than round wires [4, 18]. Studies showed that an increase in wire diameter can also increase friction [4]. Friction behavior is also influenced by the material of brackets, their dimensions such as width and interbracket span [28], and design. Ceramic brackets cause higher friction than steel brackets [7] and narrow brackets generate higher friction than wider ones [45].

This study focused on friction behavior of Gummetal® in the binding modus with a systematic comparison with other wire materials. Binding is one component of the occurring friction. Friction can be compared based on its relationship with angulation. By incrementally increasing angulation, three phases are evident [28]: (1) ligature-induced friction resulting in the lowest percentage of friction values (“classic friction”), (2) binding, and (3) notching [28, 31]. At low angulation, gliding takes place, which is restricted solely by the friction coefficient and the normal force resulting from the ligation that presses the wire into the slot. If the bracket slot and the wire become angulated to each other, the wire comes into contact with the slot edges and, as a result, elastic deformation occurs and binding adds to “classical friction” [8, 11, 42]. This critical contact angle is controlled by geometry such as the wire size, the bracket slot, the bracket width, and the interbracket distance. Binding increases with increasing contact angle and restricts sliding, until notching occurs making sliding impossible. The gliding of the tooth on the wire stops [5, 28, 31]. To minimize friction, the optimum angulation should be as little as possible beyond the angle at which the ligature-induced friction merges into binding and the angulation at which notching occurs should be avoided. These conditions are difficult to replicate in clinical practice. However, the friction in the binding phase is decisive in clinical practice and, thus, is the subject of this research.

Force application in the wire–bracket–ligature–tooth system results in complex biomechanical relationships. Teeth are tilting, rotating, and uprighting. Mastication forces also act on teeth and wires [37]. The result is that teeth are not continuously pulled along the wire and, thus, continuous sliding does not take place [16, 18]. The inconsistent movements resulting from friction between the wire and the bracket exhibit elements of both static and dynamic friction. Thus, both types of friction need to be measured. In the majority of cases, however, only the dynamic friction is measured because the complicated process of tooth movement along the wire is difficult to simulate under test conditions. Moreover, research showed that materials do not differ in ranking according to the level of their dynamic and static friction component [18]—the static proved to be higher than the kinetic friction [43]. There have been a few studies in recent years that focused on static friction [27, 35] or include measurements of static friction [43]. However, we thought that focusing on the measurement of dynamic friction is a more suitable basis for comparison of friction as it is easier to reproduce, even if clinical conditions are insufficiently simulated [16].

We looked into the following four topics:

1. How does Gummetal® perform compared to other wire materials in combination with selected brackets and slot sizes? Is its friction comparable to stainless steel?
2. How do cross-sections and wire dimensions of Gummetal® influence friction?
3. How do selected bracket types perform in combination with Gummetal® wires?
4. How does slot size (in the following slot size refers to slot height) influence the friction behavior of Gummetal® wires?

## Materials and methods

### Tested materials

To evaluate the friction values of Gummetal® and compare them to other commonly used materials, wires made of CoCr (Elgiloy®, Rocky Mountain Orthodontics, Denver, CO, USA), β‑Ti (TMA®), NiTi (NiTi-SE, Dentalline, Birkenfeld, Germany), and stainless steel were selected for this study. In order to judge the effect of wire sizes on friction, four wire dimensions including 0.014 inch (0.35 mm), 0.016 inch (0.40 mm), 0.016 × 0.022 inch (0.40 × 0.56  mm), and 0.019 × 0.025 inch (0.48 × 0.64 mm) in rod form from each of the aforementioned wire materials were applied (Table 1). However, the β‑Ti dimension 0.014 inch was not available for our research.

**Table 1:** Wire materials, manufacturers, and dimensions Drahtmaterialien, Hersteller und Dimensionen

Trade name	Manufacturer	Dimension (inch)
Gummetal®	Maruemu Works Co, Osaka, Japan/ Rocky Mountain Morita Corporation, Tokyo, Japan	0.014, 0.016, 0.016 × 0.022, 0.019 × 0.025
Elgiloy®	Rocky Mountain Orthodontics, Denver, CO, USA, distributed by Dentalline Orthodontic Products, Birkenfeld, Germany	0.014, 0.016, 0.016 × 0.022, 0.019 × 0.025
TMA®	Ormco Corp., Orange, CA, USA	0.016, 0.016 × 0.022, 0.019 × 0.025
NiTi-SE	Dentalline Orthodontic Products, Birkenfeld, Germany	0.014, 0.016, 0.016 × 0.022, 0.019 × 0.025
Stainless steel (Ref. 251–925)	3M Unitek, Monrovia, CA, USA	0.014, 0.016, 0.016 × 0.022, 0.019 × 0.025

The wires were combined with exemplary conventional brackets. Two ceramic brackets, Clarity™ (with a metal slot; 3M Unitek, Monrovia, CA, USA) and Inspire Ice™ (Ormco Corp., Orange, CA, USA) along with Discovery® (Dentaurum, Ispringen, Germany) and Micro Sprint® steel brackets (Forestadent, Pforzheim, Germany) were selected. Each bracket was tested with slot sizes of 0.018 inch (0.46 mm) and 0.022 inch (0.56 mm; Table 2). Roth prescriptions were used for all brackets except for Clarity™, which used MBT. To unify measurements, only upper right canine brackets were used. Brackets differed considerably in width. Clarity™ featured 3.7 mm, Discovery® 3.5 mm, Inspire Ice™ 3.2 mm, and Micro Sprint® 2.6 mm.

**Table 2:** Bracket systems, manufacturers, materials, and slot sizes Bracketsysteme, Hersteller, Materialien und Slotgrößen

Trade name	Manufacturer	Material	Slot size (inch)
Discovery®	Dentaurum, Ispringen, Germany	Metal	0.018 0.022
Micro Sprint®	Forestadent, Pforzheim, Germany	Metal	0.018 0.022
Clarity™	3M Unitek, Monrovia, CA, USA	Ceramic with metal slot	0.018 0.022
Inspire Ice™	Ormco Corp., Orange, CA, USA	Ceramic	0.018 0.022

Wires were ligated to the brackets with a short pretwisted 0.010 inch (0.25 mm) steel ligature (Smile Dental, Ratingen, Germany). We reduced ligature-induced friction by retwisting the ligature between 90° and 180° leaving a little play between the ligatures and the wires [40] to minimize ligature-related friction [17, 44] and allow free movement of the brackets along the wires.

### Simulation system

**Fig. 2:**
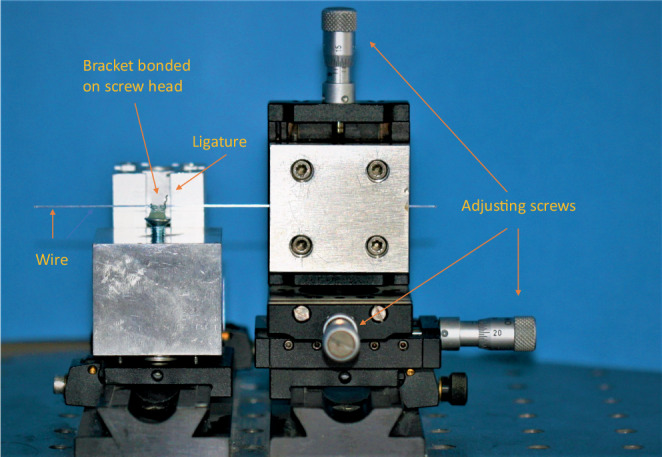
Positioning apparatus Positionierungsapparatur

**Fig. 3:**
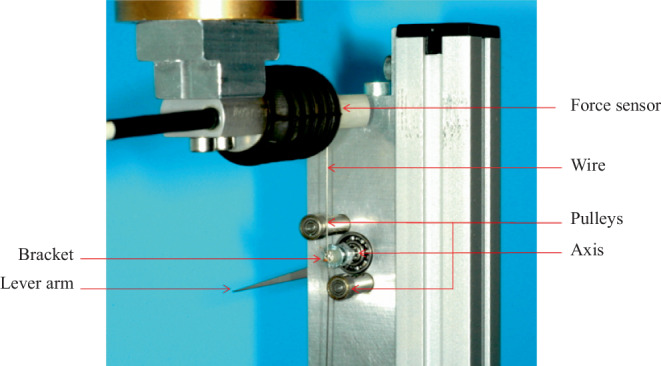
Measurement system Messsystem

For the measurement procedures, the bracket bases were bonded on flat heads of commercially available screws. We placed the brackets with an inserted slot-filling rectangular wire made of steel horizontally in a special apparatus for the bracket alignment in order to enable exact positioning (Fig. 2). The wire was held in place by stainless steel ligatures (Smile Dental, Ratingen, Germany). The ligatures were tightly applied to the wire in order to avoid play between the bracket and the wire. The apparatus was adjusted so that the bracket slots were in the center of the screws’ longitudinal axis. The bracket slots and screw heads were placed parallel to each other. In this way, influences of inset, offset, torque, and angulation of the brackets were excluded. Bracket bases and screw heads were coated with light-curing Adhesive Primer Transbond™ XT and after appropriate positioning bonded together with Light Cure Adhesive Transbond™ LR (3M Unitek, Monrovia, CA, USA). Measurements of friction between wires and brackets were made using a biomechanical measurement system (Fig. 3). Details of the setup and function can be found in previous publications [23, 39]. The measurement system consisted of the Precision Robot RX 60 Stäubli (Tec-Systems, Bayreuth, Germany) with a level of repetitive accuracy of ± 0.02 mm, the Force Sensor Type 8511-5010 (Burster Präzisionstechnik, Gernsbach, Germany), which measured the friction between the bracket and wire, and the Displacement Sensor DC/DC Series 87245 (Burster Präzisionstechnik, Gernsbach, Germany). A ball-bearing with a rotating axis was beneath the force sensor. The screws were fixed on one side of the axis. The brackets were bonded to the screws. A balance weight was fixed to a lever arm on the opposite side. In order to simulate angulation on the bracket, which usually takes place during sliding, the lever arm was loaded with a weight to generate an angulation moment of 10 Nmm. Two pulleys simulated adjacent teeth. When the wire was sliding through the bracket slot, the friction was measured by the force sensor.

### Measurements

The wire materials were tested in four different dimensions in combination with four bracket types with two different slot sizes except the 0.019 × 0.025 inch wires which did not fit into the 0.018 inch slots. The 0.014 inch β‑Ti wire was not available for testing. This resulted in 132 bracket–wire combinations being tested at room temperature and in a dry state. Testing NiTi wires at room temperature was estimated to be acceptable as they were tested at low deflection that should not have induced major martensitic transformation. All measurements were performed under the same conditions.

For each bracket–wire combination, two measurements were made. An initial measurement examined the ligature-induced friction and the friction caused by possible positioning errors (classical friction). It was made without weight on the lever arm, i.e., without applying torque or angulation. The measurement system pulled the wire, which was inserted straight into the bracket slot, for 40 mm at a constant speed of 0.2 mm/s through the bracket slot. The force registered by the sensor was then sent to the measuring system computer program. The sampling frequency was set at 10 Hz. During the almost 4 min test period, 2000 measurements were made. The first and the last measurements were discarded to exclude static friction at the beginning and end of the movement, since only dynamic friction was to be evaluated. Thus, 1942 values remained for further evaluation. The second measurement used the angulation weight on the lever arm using otherwise identical measuring conditions. A total of 1942 measurements were taken from which the values of the blank measurements were discarded, as they were not relevant for examining friction in the binding modus.

**Fig. 4:**
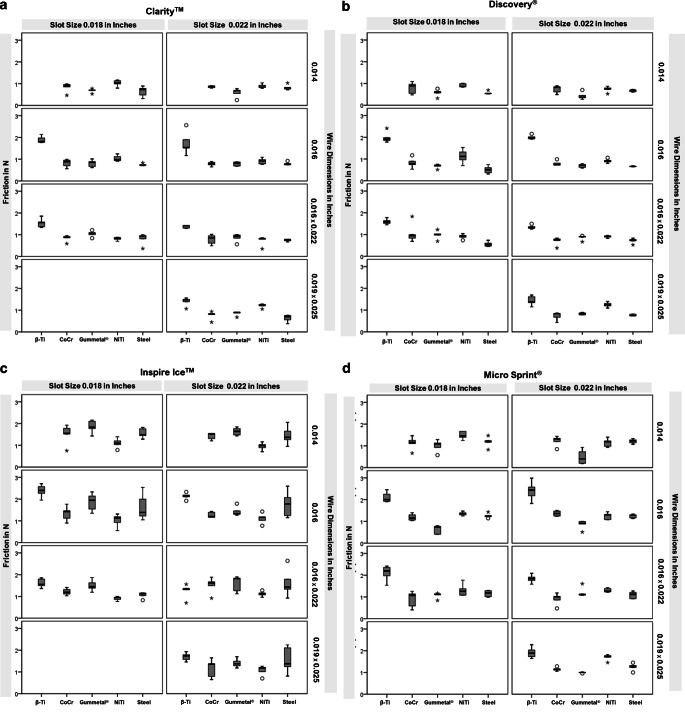
Friction (in N) of **a** Clarity™, **b** Discovery®, **c** Inspire Ice™, and **d** Micro Sprint® in combination with tested wire materials and dimensions presented in box plots. The *box* covers 50% of the data from 25–75 percentiles. The *line* within the box marks the median, the *whiskers* mark the minimum and maximum, the *circles* the outliers and the *asterisks* the “extreme” outliers Friktion (N) von **a** Clarity™, **b **Discovery®, **c** Inspire Ice™ und **d** Micro Sprint® in Kombination mit getesteten Drahtmaterialien und -dimensionen dargestellt in Box Plots. Die *Box* umfasst 50% der Daten von der 25. bis zur 75. Perzentile. Die *Linie* innerhalb der Box markiert den Median, *Whiskers* markieren Minima und Maxima, *Kreise* Ausreißer, *Sternchen* „extreme“ Ausreißer

We performed five identical tests for each of the 132 bracket–wire combinations and used a new bracket for each measurement in order to ensure the original properties of the brackets**. **The mean values were calculated yielding in a total of 660 mean values, five for each bracket–wire combination. Again, mean values were calculated for each of these five combinations. These mean values, including the respective minima and maxima, medians, and standard deviations for the 132 bracket–wire combinations were arranged according to the tested bracket type and are presented in Table 3. Box plots are shown in Fig. 4. The values were then statistically evaluated.

**Table 3:** Descriptive statistics: Test results for the 132 bracket–wire combinations arranged according to the tested bracket type: Clarity™, Discovery®, Inspire Ice™, and Micro Sprint®. Number of tests per bracket–wire combination (N), medians (MD), mean values (M), standard deviations (SD), minima (Min), and maxima (Max) each in Newton Deskriptive Statistik: Testergebnisse für die 132 Bracket-Draht-Kombinationen in der Reihenfolge der getesteten Brackettypen: Clarity™, Discovery®, Inspire Ice™ und Micro Sprint®. Zahl der Tests je Bracket-Draht-Kombination (N), Mediane (MD), Mittelwerte (M), Standardabweichungen (SD), Minima (Min) und Maxima (Max) jeweils in Newton

Group	*N*	MD	M	SD	Min	Max
*Clarity™*						
Clarity™ 18, β‑Ti 16	5	1.819	1.894	0.160	1.763	2.138
Clarity™ 18, β‑Ti 16 × 22	5	1.565	1.539	0.206	1.338	1.852
Clarity™ 18, CoCr 14	5	0.903	0.833	0.212	0.464	0.979
Clarity™ 18, CoCr 16	5	0.887	0.819	0.182	0.560	0.995
Clarity™ 18, CoCr 16 × 22	5	0.897	0.843	0.147	0.588	0.955
Clarity™ 18, Gummetal® 14	5	0.698	0.679	0.089	0.533	0.775
Clarity™ 18, Gummetal® 16	5	0.856	0.809	0.163	0.618	1.012
Clarity™ 18, Gummetal® 16 × 22	5	1.079	1.043	0.135	0.841	1.210
Clarity™ 18, NiTi 14	5	1.043	1.025	0.145	0.798	1.164
Clarity™ 18, NiTi 16	5	0.977	1.038	0.141	0.910	1.246
Clarity™ 18, NiTi 16 × 22	5	0.827	0.817	0.074	0.700	0.888
Clarity™ 18, Stahl 14	5	0.735	0.640	0.236	0.313	0.888
Clarity™ 18, Stahl 16	5	0.735	0.744	0.053	0.699	0.834
Clarity™ 18, Stahl 16 × 22	5	0.938	0.815	0.263	0.362	1.004
Clarity™ 22, β‑Ti 16	5	1.534	1.737	0.529	1.168	2.563
Clarity™ 22, β‑Ti 16 × 22	5	1.413	1.369	0.066	1.289	1.421
Clarity™ 22, β‑Ti 19 × 25	5	1.483	1.398	0.194	1.067	1.559
Clarity™ 22, CoCr 14	5	0.871	0.857	0.044	0.800	0.903
Clarity™ 22, CoCr 16	5	0.784	0.782	0.093	0.646	0.886
Clarity™ 22, CoCr 16 × 22	5	0.853	0.783	0.223	0.490	1.011
Clarity™ 22, CoCr 19 × 25	5	0.822	0.771	0.186	0.451	0.938
Clarity™ 22, Gummetal® 14	5	0.679	0.586	0.206	0.246	0.768
Clarity™ 22, Gummetal® 16	5	0.819	0.787	0.098	0.675	0.880
Clarity™ 22, Gummetal® 16 × 22	5	0.937	0.866	0.182	0.560	1.005
Clarity™ 22, Gummetal® 19 × 25	5	0.896	0.851	0.098	0.677	0.907
Clarity™ 22, NiTi 14	5	0.842	0.884	0.100	0.786	1.039
Clarity™ 22, NiTi 16	5	0.915	0.906	0.137	0.761	1.092
Clarity™ 22, NiTi 16 × 22	5	0.814	0.729	0.213	0.349	0.852
Clarity™ 22, NiTi 19 × 25	5	1.225	1.206	0.091	1.052	1.286
Clarity™ 22, Stahl 14	5	0.817	0.829	0.118	0.725	1.028
Clarity™ 22, Stahl 16	5	0.767	0.793	0.075	0.739	0.921
Clarity™ 22, Stahl 16 × 22	5	0.773	0.748	0.049	0.671	0.784
Clarity™ 22, Stahl 19 × 25	5	0.701	0.631	0.163	0.378	0.764
*Discovery®*						
Discovery® 18, β‑Ti 16	5	1.911	1.988	0.248	1.780	2.412
Discovery® 18, β‑Ti 16 × 22	5	1.582	1.593	0.126	1.448	1.778
Discovery® 18, CoCr 14	5	0.896	0.794	0.267	0.478	1.088
Discovery® 18, CoCr 16	5	0.786	0.824	0.233	0.533	1.170
Discovery® 18, CoCr 16 × 22	5	0.952	1.059	0.447	0.688	1.831
Discovery® 18, Gummetal® 14	5	0.614	0.576	0.161	0.318	0.759
Discovery® 18, Gummetal® 16	5	0.690	0.673	0.095	0.511	0.755
Discovery® 18, Gummetal® 16 × 22	5	1.009	0.986	0.191	0.694	1.227
Discovery® 18, NiTi 14	5	0.867	0.910	0.082	0.841	1.012
Discovery® 18, NiTi 16	5	1.118	1.126	0.319	0.699	1.528
Discovery® 18, NiTi 16 × 22	5	0.920	0.908	0.112	0.737	1.039
Discovery® 18, Steel 14	5	0.540	0.565	0.070	0.522	0.690
Discovery® 18, Steel 16	5	0.505	0.506	0.172	0.313	0.741
Discovery® 18, Steel 16 × 22	5	0.545	0.561	0.113	0.452	0.735
Discovery® 22, β‑Ti 16	5	1.970	1.999	0.096	1.920	2.157
Discovery® 22, β‑Ti 16 × 22	5	1.308	1.344	0.091	1.253	1.492
Discovery® 22, β‑Ti 19 × 25	5	1.375	1.435	0.218	1.150	1.692
Discovery® 22, CoCr 14	5	0.830	0.734	0.174	0.491	0.896
Discovery® 22, CoCr 16	5	0.744	0.797	0.113	0.721	0.988
Discovery® 22, CoCr 16 × 22	5	0.745	0.696	0.182	0.378	0.830
Discovery® 22, CoCr 19 × 25	5	0.836	0.729	0.185	0.428	0.867
Discovery® 22, Gummetal® 14	5	0.366	0.429	0.164	0.271	0.699
Discovery® 22, Gummetal® 16	5	0.710	0.689	0.085	0.588	0.789
Discovery® 22, Gummetal® 16 × 22	5	0.903	0.862	0.111	0.666	0.938
Discovery® 22, Gummetal® 19 × 25	5	0.849	0.835	0.049	0.783	0.895
Discovery® 22, NiTi 14	5	0.761	0.736	0.129	0.525	0.871
Discovery® 22, NiTi 16	5	0.912	0.915	0.094	0.802	1.055
Discovery® 22, NiTi 16 × 22	5	0.919	0.905	0.052	0.825	0.960
Discovery® 22, NiTi 19 × 25	5	1.251	1.249	0.115	1.094	1.400
Discovery® 22, Steel 14	5	0.687	0.670	0.050	0.611	0.736
Discovery® 22, Steel 16	5	0.660	0.661	0.018	0.643	0.684
Discovery® 22, Steel 16 × 22	5	0.738	0.715	0.116	0.524	0.833
Discovery® 22, Steel 19 × 25	5	0.743	0.769	0.038	0.741	0.811
*Inspire Ice™*						
Inspire Ice™ 18, β‑Ti 16	5	2.399	2.380	0.292	1.953	2.708
Inspire Ice™ 18, β‑Ti 16 × 22	5	1.553	1.613	0.204	1.365	1.849
Inspire Ice™ 18, CoCr 14	5	1.546	1.504	0.452	0.752	1.924
Inspire Ice™ 18, CoCr 16	5	1.425	1.335	0.334	0.897	1.766
Inspire Ice™ 18, CoCr 16 × 22	5	1.188	1.211	0.153	1.028	1.407
Inspire Ice™ 18, Gummetal® 14	5	1.836	1.859	0.293	1.428	2.156
Inspire Ice™ 18, Gummetal® 16	5	1.945	1.860	0.407	1.349	2.329
Inspire Ice™ 18, Gummetal® 16 × 22	5	1.445	1.502	0.255	1.191	1.864
Inspire Ice™ 18, NiTi 14	5	1.100	1.101	0.221	0.786	1.389
Inspire Ice™ 18, NiTi 16	5	1.138	1.023	0.297	0.554	1.312
Inspire Ice™ 18, NiTi 16 × 22	5	0.948	0.907	0.092	0.767	0.995
Inspire Ice™ 18, Steel 14	5	1.478	1.556	0.223	1.283	1.818
Inspire Ice™ 18, Steel 16	5	1.383	1.637	0.617	1.046	2.535
Inspire Ice™ 18, Steel 16 × 22	5	1.126	1.061	0.139	0.828	1.157
Inspire Ice™ 22, β‑Ti 16	5	2.152	2.138	0.148	1.918	2.326
Inspire Ice™ 22, β‑Ti 16 × 22	5	1.337	1.254	0.325	0.700	1.558
Inspire Ice™ 22, β‑Ti 19 × 25	5	1.736	1.698	0.185	1.458	1.932
Inspire Ice™ 22, CoCr 14	5	1.519	1.424	0.158	1.203	1.549
Inspire Ice™ 22, CoCr 16	5	1.197	1.270	0.132	1.162	1.433
Inspire Ice™ 22, CoCr 16 × 22	5	1.611	1.516	0.363	0.918	1.884
Inspire Ice™ 22, CoCr 19 × 25	5	1.358	1.164	0.421	0.650	1.646
Inspire Ice™ 22, Gummetal® 14	5	1.642	1.634	0.181	1.429	1.850
Inspire Ice™ 22, Gummetal® 16	5	1.326	1.427	0.216	1.255	1.790
Inspire Ice™ 22, Gummetal® 16 × 22	5	1.823	1.587	0.364	1.120	1.894
Inspire Ice™ 22, Gummetal® 19 × 25	5	1.370	1.405	0.201	1.182	1.703
Inspire Ice™ 22, NiTi 14	5	0.988	0.956	0.169	0.707	1.162
Inspire Ice™ 22, NiTi 16	5	1.167	1.113	0.238	0.775	1.426
Inspire Ice™ 22, NiTi 16 × 22	5	1.130	1.119	0.116	0.959	1.278
Inspire Ice™ 22, NiTi 19 × 25	5	1.180	1.073	0.228	0.701	1.264
Inspire Ice™ 22, Steel 14	5	1.371	1.457	0.417	0.952	2.055
Inspire Ice™ 22, Steel 16	5	1.773	1.764	0.600	1.143	2.593
Inspire Ice™ 22, Steel 16 × 22	5	1.428	1.621	0.643	0.926	2.632
Inspire Ice™ 22, Steel 19 × 25	5	1.376	1.555	0.604	0.809	2.242
*Micro Sprint®*						
Micro Sprint® 18, β‑Ti 16	5	2.005	2.107	0.240	1.901	2.459
Micro Sprint® 18, β‑Ti 16 × 22	5	2.193	2.096	0.356	1.538	2.415
Micro Sprint® 18, CoCr 14	5	1.156	1.129	0.295	0.663	1.467
Micro Sprint® 18, CoCr 16	5	1.154	1.189	0.155	1.037	1.397
Micro Sprint® 18, CoCr 16 × 22	5	1.074	0.892	0.373	0.405	1.259
Micro Sprint® 18, Gummetal® 14	5	1.091	1.002	0.271	0.574	1.288
Micro Sprint® 18, Gummetal® 16	5	0.744	0.627	0.201	0.407	0.811
Micro Sprint® 18, Gummetal® 16 × 22	5	1.119	1.074	0.132	0.840	1.166
Micro Sprint® 18, NiTi 14	5	1.454	1.485	0.180	1.246	1.666
Micro Sprint® 18, NiTi 16	5	1.369	1.356	0.084	1.250	1.472
Micro Sprint® 18, NiTi 16 × 22	5	1.266	1.315	0.284	1.079	1.771
Micro Sprint® 18, Steel 14	5	1.215	1.182	0.233	0.823	1.471
Micro Sprint® 18, Steel 16	5	1.245	1.259	0.106	1.141	1.430
Micro Sprint® 18, Steel 16 × 22	5	1.183	1.156	0.146	0.994	1.299
Micro Sprint® 22, β‑Ti 16	5	2.441	2.401	0.438	1.820	2.991
Micro Sprint® 22, β‑Ti 16 × 22	5	1.838	1.836	0.185	1.588	2.088
Micro Sprint® 22, β‑Ti 19 × 25	5	1.888	1.913	0.251	1.643	2.275
Micro Sprint® 22, CoCr 14	5	1.320	1.230	0.228	0.851	1.437
Micro Sprint® 22, CoCr 16	5	1.362	1.364	0.125	1.233	1.505
Micro Sprint® 22, CoCr 16 × 22	5	1.008	0.909	0.269	0.474	1.185
Micro Sprint® 22, CoCr 19 × 25	5	1.160	1.154	0.086	1.054	1.285
Micro Sprint® 22, Gummetal® 14	5	0.400	0.486	0.330	0.176	0.925
Micro Sprint® 22, Gummetal® 16	5	0.911	0.859	0.202	0.508	1.000
Micro Sprint® 22, Gummetal® 16 × 22	5	1.103	1.205	0.226	1.084	1.609
Micro Sprint® 22, Gummetal® 19 × 25	5	0.999	0.996	0.024	0.957	1.016
Micro Sprint® 22, NiTi 14	5	1.185	1.145	0.189	0.935	1.396
Micro Sprint® 22, NiTi 16	5	1.302	1.247	0.158	1.082	1.443
Micro Sprint® 22, NiTi 16 × 22	5	1.268	1.306	0.094	1.216	1.425
Micro Sprint® 22, NiTi 19 × 25	5	1.746	1.699	0.142	1.456	1.820
Micro Sprint® 22, Steel 14	5	1.229	1.211	0.105	1.072	1.338
Micro Sprint® 22, Steel 16	5	1.289	1.243	0.089	1.132	1.333
Micro Sprint® 22, Steel 16 × 22	5	1.119	1.087	0.165	0.912	1.274
Micro Sprint® 22, Steel 19 × 25	5	1.277	1.255	0.164	1.001	1.450

## Statistical analysis

Statistical analysis was conducted using the SPSS® (Version 22, IBM, Armonk, NY, USA) to determine whether friction of different wire sizes of Gummetal®, in combination with the tested brackets and slot sizes, was significantly different from the friction of the other analyzed wires. The five mean values of each of the bracket–wire combinations were examined for normal distribution using the Shapiro–Wilk test. Nonparametric Kruskal–Wallis tests were chosen for intergroup comparisons (*p* < 0.05) since in 14 of the 132 combinations no normal distribution could be found (*p* < 0.05). It was followed by nonparametric Mann–Whitney U tests for pair comparisons between Gummetal® and the other wire materials of the same dimensions in combination with the selected brackets and slot sizes analyzing whether significant differences for friction were present (*p* < 0.05, Bonferroni-corrected).

The statistical procedure for analyzing the influence of cross-sections and wire dimensions on the friction behavior of Gummetal® corresponded to question 1. We used Kruskal–Wallis tests to test whether significant differences (*p* < 0.05) were observed within the Gummetal® groups and Mann–Whitney U tests for pair comparisons. This analyzed whether the differences between the friction of different cross-sections and dimensions of Gummetal® in combination with the tested brackets and slot sizes were significant (*p* < 0.05, Bonferroni corrected).

Regarding friction of the different types of brackets and slot sizes in combination with the four wire dimensions of Gummetal®, Kruskal–Wallis tests were used in order to determine if there were significant differences (*p* < 0.05) within the groups of different types of brackets with respect to slot size and wire dimension. (Dimension 0.019 × 0.025 inch did not fit into the 0.018 inch slots.) This was followed by Mann–Whitney U tests for pair comparisons to determine the level of significance of the friction variances between the different types of brackets of the same slot height combined with different sizes of Gummetal® (*p* < 0.05, Bonferroni-corrected).

We examined the differences in friction of the two slot sizes (0.018 inch and 0.022 inch) of each type of bracket in combination with the Gummetal® wires in the dimensions 0.014 inch, 0.016 inch, and 0.016 × 0.022 inch using Mann–Whitney U tests (*p* < 0.05).

## Results

The results of pair comparisons using Mann–Whitney U tests are presented in Table 4 classifying differences of friction as significant (^*^) or not significant (n. s.). Differences were found to be significant in 37 cases out of a total of 104 pair comparisons.

**Table 4:** Mann–Whitney U tests: Gummetal® with NiTi, β‑Ti, stainless steel and CoCr. The results of pair comparisons between Gummetal® wires and other wire alloys with same dimensions combined with different brackets and slot sizes are presented. Friction differences significant (*) or not significant (*n.s.*) Mann-Whitney-U-Tests: Gummetal® mit NiTi, β‑Ti, Edelstahl und CoCr. Dargestellt sind die Ergebnisse der gezielten Paarvergleiche zwischen Gummetal®-Drähten und jeweils einer weiteren Drahtlegierung derselben Dimension in Verbindung mit unterschiedlichen Brackets und Slotgrößen. Friktionsunterschiede signifikant (*) bzw. nicht signifikant (*n.s.*)

Bracket Gummetal®	NiTi	β‑Ti	Stainlesss steel	CoCr
Clarity™ 18 Dim. 14	^*^(< 0.0125)		n. s. (0.841)	n. s. (0.151)
Clarity™ 18 Dim. 16	n. s. (0.056)	^*^ (< 0.0125)	n. s. (0.69)	n. s. (1.00)
Clarity™ 18 Dim. 16 × 22	n. s. (0.032)	^*^ (< 0.0125)	n. s. (0.09)	n. s. (0.095)
Clarity™ 22 Dim. 14	^*^(< 0.0125)		n. s. (0.032)	^*^(< 0.0125)
Clarity™ 22 Dim. 16	n. s. (0.222)	^*^ (< 0.0125)	n. s. (1.00)	n. s. (1.00)
Clarity™ 22 Dim. 16 × 22	n. s. (0.151)	^*^ (< 0.0125)	n. s. (0.151)	n. s. (0.841)
Clarity™ 22 Dim. 19 × 25	^*^(< 0.0125)	^*^ (< 0.0125)	n. s. (0.056)	n. s. (0.421)
Discovery® 18 Dim. 14	^*^(< 0.0125)		n. s. (0.421)	n. s. (0.421)
Discovery® 18 Dim. 16	n. s. (0.032)	^*^ (< 0.0125)	n. s. (0.151)	n. s. (0.151)
Discovery® 18 Dim. 16 × 22	n. s. (0.421)	^*^(< 0.0125)	n. s. (0.016)	n. s. (0.548)
Discovery® 22 Dim. 14	n. s. (0.016)		n. s. (0.095)	n. s. (0.032)
Discovery® 22 Dim. 16	^*^(< 0.0125)	^*^(< 0.0125)	n. s. (0.69)	n. s. (0.222)
Discovery® 22 Dim. 16 × 22	n. s. (0.69)	^*^(< 0.0125)	n. s. (0.095)	n. s. (0.095)
Discovery® 22 Dim. 19 × 25	^*^(< 0.0125)	^*^(< 0.0125)	n. s. (0.095)	n. s. (0.421)
Inspire Ice™ 18 Dim. 14	^*^(< 0.0125)		n. s. (0.151)	n. s. (0.31)
Inspire Ice™ 18 Dim. 16	^*^(< 0.0125)	n. s. (0.056)	n. s. (0.548)	n. s. (0.095)
Inspire Ice™ 18 Dim. 16 × 22	^*^(< 0.0125)	n. s. (0.69)	^*^(< 0.0125)	n. s. (0.056)
Inspire Ice™ 22 Dim. 14	^*^(< 0.0125)		n. s. (0.421)	n. s. (0.222)
Inspire Ice™ 22 Dim. 16	n. s. (0.056)	^*^(< 0.0125)	n. s. (0.841)	n. s. (0.222)
Inspire Ice™ 22 Dim. 16 × 22	n. s. (0.095)	n. s. (0.421)	n. s. (0.841)	n. s. (0.69)
Inspire Ice™ 22 Dim. 19 × 25	n. s. (0.032)	n. s. (0.056)	n. s. (0.841)	n. s. (0.421)
Micro Sprint® 18 Dim. 14	n. s. (0.016)		n. s. (0.31)	n. s. (0.421)
Micro Sprint® 18 Dim. 16	^*^(< 0.0125)	^*^(< 0.0125)	^*^(< 0.0125)	^*^(< 0.0125)
Micro Sprint® 18 Dim. 16 × 22	n. s. (0.421)	^*^(< 0.0125)	n. s. (0.421)	n. s. (0.69)
Micro Sprint® 22 Dim. 14	^*^(< 0.0125)		^*^(< 0.0125)	n. s. (0.016)
Micro Sprint® 22 Dim. 16	^*^(< 0.0125)	^*^(< 0.0125)	^*^(< 0.0125)	^*^(< 0.0125)
Micro Sprint® 22 Dim. 16 × 22	n. s. (0.151)	n. s. (0.016)	n. s. (0.841)	n. s. (0.095)
Micro Sprint® 22 Dim. 19 × 25	^*^(< 0.0125)	^*^(< 0.0125)	n. s. (0.032)	^*^(< 0.0125)

*p* < 0.05 (Bonferroni-corrected: *p* < 0.0125) (*p*-value)

Empty field not tested

*Dim.* Dimension

These results were combined with the findings of the measured friction of the 132 groups of bracket–wire combinations and are presented in Table 3 and Fig. 4.

Thus, regarding question 1, we concluded that Gummetal® was, in most combinations, comparable with stainless steel. In three out of seven combinations with the Micro Sprint® bracket, friction was significantly lower than that of stainless steel (mean value of friction for Gummetal® versus steel: 0.627 N versus 1.259 N for slot size 0.018 inch and wire dimension 0.016 inch; 0.486 N versus 1.211 N for slot size 0.022 inch and wire dimension 0.014 inch; 0.859 N versus 1.243 N for slot size 0.022 inch and wire dimension 0.016 inch). It was only for the dimension 0.016 × 0.022 inch in combination with the ceramic bracket Inspire Ice™ at slot size 0.018 inch that friction was significantly higher for Gummetal® than for stainless steel (mean value of friction for Gummetal® versus steel: 1.502 N versus 1.061 N). In combination with the Discovery® bracket at slot size 0.022 inch, Gummetal® of the dimension 0.014 inch had the lowest mean value (0.429 N), the lowest median (0.366 N), and the lowest maximum value (0.699 N) of all combinations tested.

Compared to CoCr wires, also considered to have low friction, the friction of Gummetal® was never significantly higher. In four combinations it was significantly lower, among them three combinations with the Micro Sprint® bracket (mean value of friction for Gummetal® versus CoCr: 0.627 N versus 1.189 N for slot size 0.018 inch and wire dimension 0.016 inch; 0.859 N versus 1.364 N for slot size 0.022 inch and wire dimension 0.016 inch; 0.996 N versus 1.154 N for slot size 0.022 inch and wire dimension 0.019 × 0.025 inch). The friction of Gummetal® was in 10 out of 28 combinations significantly lower compared with NiTi wires. In four combinations with the Inspire Ice™ bracket, friction values of Gummetal® were significantly higher than those of NiTi. When NiTi was combined with Inspire Ice™ brackets, it showed a better performance than Gummetal® and stainless steel. Compared with β‑Ti, the friction of Gummetal® was significantly lower in 15 out of 20 combinations (e.g., the mean value of friction of Gummetal® versus β‑Ti in combination with the Micro Sprint bracket was 0.627 N versus 2.107 N for slot size 0.018 inch and wire dimension 0.016 inch).

Looking into question 2 about how cross-sections and wire dimensions of Gummetal® influence its friction, we found that three out of eight combinations of the round wires with brackets slot size 0.018 inch demonstrated significantly lower friction than rectangular 0.016 × 0.022 inch wires. Differences of friction between round and rectangular wires in combination with brackets of slot size 0.022 inch, between the two dimensions of round wires, and between the two dimensions of rectangular wires were not significant. Mean values of friction of round wires were generally lower than those of rectangular wires in combination with most brackets except for the Inspire Ice™ bracket.

Considering question 3, there was no significant difference for friction between all brackets combined with Gummetal® wires. In this test series, the level of significance, adjusted by Bonferroni correction, was as low as *p* < 0.008. We observed a few remarkable differences in the mean values of friction for some bracket–wire combinations. For example, the mean value for Gummetal® dimension 0.014 inch with slot size 0.018 inch was 0.576 N in combination with the Discovery® bracket and 1.859 N in combination with the Inspire Ice™ bracket. Furthermore, the mean value for Gummetal® dimension 0.016 inch slot size 0.018 inch was 0.627 N in combination with the Micro Sprint® bracket and 1.860 N in combination with Inspire Ice™ bracket.

Regarding question 4, the slot size did not significantly affect the friction in 9 of 12 pair comparisons. Significantly less friction resulted in one combination with the Micro Sprint® bracket and in one combination with the Inspire Ice™ bracket when using higher slots.

## Discussion

As the amount of friction of Gummetal® was found to be comparable to that of other low-friction wires, the low friction force could be explained by its low Young’s modulus of about 45 GPa [22]. This is higher than the Young’s modulus of NiTi with 33–44 GPa and lower than that of β‑Ti with approximately 72 GPa [29]. However, stainless steel and CoCr have significantly higher Young’s modules ranging from 160–180 GPa and from 160–190 GPa [25] but very low friction. Thus, it cannot be assumed that there is a connection between Young’s modules of the respective wire alloys and friction in binding mode. Frictional differences must be caused by other material properties, e.g., the surface structure [16].

In Fig. 1, the images of the surface structure of Gummetal® and the other tested wires are presented. The surface roughness of materials is often quantified on the basis of surface profilometry, laser specular reflection or other techniques by reducing all of the information in a profile to a single number (Ra, RMS, etc.) [26]. At the time of this research, those methods applied for measuring the surface roughness of the other wires, as presented e.g. by Bourauel et al. [10], were not used for assessing the surface roughness of Gummetal®. Should the “optical” appearance of Gummetal®, which seemed less smooth than that of steel, be confirmed by roughness parameters, the low friction of Gummetal® may be considered an anomaly. Thus, Gummetal®’s low friction would require further explanation, as other β‑Ti alloys usually demonstrate high friction.

It has been found that the friction of materials with an apparently smooth surface tends to be high if production-related burrs protrude into the slot [16] or if the archwires show structural irregularities [20]. Thus, the geometry of irregularities of wire surfaces should be investigated further. Moreover, the “marble like” surface structure and structural nonuniformities of Gummetal® [38] consisting of four components (titanium, niobium, tantalum, and zirconium) may also explain its low friction which could be examined by looking into the classical “surface surface friction” of Gummetal® in future research. These nonuniformities may also explain the anomalous mechanical properties of Gummetal® such as its nonlinear elastic behavior [33].

Our results corroborated the observation that wire dimension influences the friction of bracket–wire systems, i.e., that rectangular wires produce higher friction than round ones and friction increases when diameter increases [16].

The friction values of Gummetal® wires when combined with selected bracket types were comparable to those described in other tests, for example, tests of ceramic brackets [13, 41]. All of these differences were not significant, but the monocrystalline ceramic brackets Inspire Ice™ yielded much higher friction values—up to three times higher—than the other brackets in this study. Since this bracket type is usually known to have a smooth surface, the high friction generated by Inspire Ice™ may have been caused by the sharp edges of its slots. The polycrystalline ceramic bracket Clarity™, unlike other polycrystalline ceramic brackets [1] assessed in studies to date, produced less friction than Inspire Ice™. Its friction behavior was similar to that of steel brackets possibly because it has metal slots making it comparable to steel brackets such as Discovery®.

The effects of width differences of the bracket slots were less clear in our findings. Friction typically behaves inversely proportional to bracket width [19], as the effective force on the edges of the slot is higher in narrow brackets than in wider ones. Thus, the smallest of our tested brackets, Micro Sprint®, should go together with higher friction compared with the other tested brackets. A few results of our tests of different bracket types in combination with Gummetal® wires supported this connection, others did not. The mean value of friction of the Discovery® 0.018 inch bracket in combination with Gummetal® 0.014 inch, for instance, was 0.576 N, while that of the Micro Sprint® 0.018 inch bracket, in the same combination, was as high as 1.002 N. In some combinations, the mean value of friction of the Micro Sprint® bracket was even lower than that of the Discovery® bracket. In these cases, the theoretical favorable friction properties of wider brackets may have been compensated by other factors such as unfavorable surface structures, slot edges, or deviations in dimensions [12, 16].

We found that the slot heights in most combinations showed no significant influence on friction which is consistent with the results of other studies [39]. There is, however, a problem when comparing friction for different bracket types since there can be significant production inherent variability in size of an individual bracket. For instance, the Discovery® bracket showed a size variability of up to 24% [12]. We also observed significant variability in slot height of the Discovery® brackets in the same batch. Thus, results from friction measurements of individual brackets should be treated with caution. Product variations are also important in the clinical practice because, for instance, oversized slots not only decrease friction values compared to the standardized ones, they may also prevent efficient tooth control [28].

### Study limitations

This study has been limited to comparisons of frequently used materials. Many other combinations still remain to be tested. For instance, we did not combine Gummetal® with self-ligating brackets. In many tests, self-ligating brackets did not show improved performance compared with conventional brackets [34]. However, different to the application of steel ligature by hand, they have not to be considered as a possible source of nonconformity in tests.

Under clinical conditions, actual friction may differ from our in vitro tests that did not capture clinical scenarios such as mastication, lubrication, physiological tooth mobility, body temperature, etc. Testing at 36 °C, for instance, might have provided a better picture of the clinical available mechanical values, at least for NiTi, which is known for the change in its mechanical behavior at different temperatures. However, the identified ranking of the archwires including Gummetal® may be helpful for practitioners.

The focus of this study has been on dynamic friction, as it is easier to simulate than static friction. Moreover, tests have shown that materials do not differ in ranking according to the level of their dynamic or static friction. But, in clinical treatment, the tooth experiences dynamic as well as static friction because of its piecewise movement, which in sum should be higher than the dynamic friction measured in our tests [16, 17]. The forces of static friction of Gummetal® will remain a topic for future studies.

## Conclusion

The friction behavior of Gummetal®, an alloy consisting of four components, was comparable to stainless steel which is considered to present low friction. Low friction is thus another advantageous characteristic of Gummetal®. This adds to the already published other merits which included the following: its elastic behavior that fills the gap between β‑Ti and NiTi and is far below that of steel; the small forces produced when deforming Gummetal® enabling 3‑dimensional control of tooth movements with rectangular wires at all stages of treatment, except at the very early stage [14]; its biocompatibility; its properties exhibiting high strength as well as good formability. Thus, we consider Gummetal® a valuable addition to other orthodontic wire materials.
